# Supplementary material for: The role of glutathione in periplasmic redox homeostasis and oxidative protein folding in Escherichia coli
Source: Redox Biol. 2023 Jun 26;64:102800. doi: 10.1016/j.redox.2023.102800 (PMC10344953; doi:10.1016/j.redox.2023.102800)
Supplement: Multimedia component 1 [file mmc1.docx]

# Supplementary material

**Supplementary table 1. Bacterial and yeast strains used in this study**.

| **Strain** | **Relevant characteristics** | **Reference** |
| --- | --- | --- |
| *E. coli* strains |  |  |
| DH5α | Cloning host | (Taylor et al., 1993) |
| BL21 (DE3) | Expression host | (Studier and Moffatt, 1986) |
| MG1655 | Expression host | (Blattner et al., 1997) |
|  |  |  |
| *E. coli* KEIO strains |  |  |
| WT (BW25113) | Δ(*araD-araB*)567, Δ*lacZ*4787(::*rrnB*-3), λ-, *rph*-1, Δ(*rhaD-rhaB*)568, *hsdR*514 | (Baba et al., 2006) |
| Δ*gshA* (JW2663) | BW25113; Δ*gshA*769::km |  |
| Δ*dsbA* (JW3832) | BW25113; Δ*dsbA*723::km |  |
| Δ*gshA*Δ*dsbA* | BW25113; Δ*gshA*; Δ*dsbA*723::km | This study |
| Δ*dsbB* (JW5182-1) | BW25113; Δ*dsbB774*::km | (Baba et al., 2006) |
|  |  |  |
| Yeast strains |  |  |
| YPH499 | Δ*glr1*Δ*grx1*Δ*grx2* | (Zimmermann et al., 2021) |
|  |  |  |

**Supplementary table 2. Plasmids used in this study**.

| **Plasmid** | **Relevant characteristics** | **Reference** |
| --- | --- | --- |
| Cloning/ template plasmids | |  |
| pPT | IPTG-inducible, pCC derivative, *torA* | This study |
| pCC | IPTG-inducible, TAC-MAT-Tag-2 derivative; p*tac,* Amp^R^ | (Degrossoli et al., 2018; Masuch et al., 2015) |
| pLK8 (pQE30-*roGFP-iL*) | roGFP-iL template, Amp^R^ | Addgene, USA, #83313 (Lohman and Remington, 2008) |
| p416TEF_roGFP2-AtPrxA-ΔCP | Yeast expression plasmid coding for AtPrxA-ΔCP, Amp^R^ | (Zimmermann et al., 2021) |
|  |  |  |
| *E. coli* expression plasmids | |  |
| pCC_*roGFP2* | pTAC-MAT-Tag-2 derivative; *roGFP2*, *ptac,* Amp^R^ | (Müller et al., 2017) |
| pPT | pTAC-MAT-Tag-2 derivative containing the N-terminal TorA TAT signal sequence | This study |
| pPT_*roGFP2* | pPT containing *roGFP2* | This study |
| pLK9 (pPT_*roGFP-iL*) | pPT containing *roGFP-iL* | This study |
| pOE1_*dsbA*ΔSP | pET11a-derivative, Strep-tag, TEV-site, *dsbA*ΔSP, Amp^R^ | Lab collection |
|  |  |  |
| Yeast expression plasmids | |  |
| pLK16 (p416TEF_roGFP2-*Ec*DsbAΔSP) | Yeast expression plasmid coding for *EcDsbA*ΔSP, Amp^R^ | This study |
| p415TEF-*OPT1* | Yeast expression plasmid coding for Opt1/Hgt1 | (Zimmermann et al., 2021) |
| p416TEF_*roGFP2* | Yeast expression plasmid coding for roGFP2 | (Zimmermann et al., 2021) |
|  |  |  |
| Removal of kanamycin cassette | |  |
| 709-*Flpe* | *flpe*, λR promoter, heat-labile cI857 repressor Amp^R^ | Gene Bridges, Germany, #A106 |

**Supplementary table 3. Oligonucleotides used in this study**. Restriction and mutagenesis sites are underlined.

| **Primer** | | **Oligonucleotide sequence (5´🡪 3´)** | |
| --- | --- | --- | --- |
| pPT plasmid for periplasmic targeting | |  |  |
| *torA_NdeI-fwd* |  | GGGGGCATATGAACAATAACGATCTCTTTCAG | |
| *torA_rev* |  | GGAATACATATGCGCCGCTTGCGCCGCAGT | |
| *QC-Nde-fw* |  | GGAGATATCGTATGAACAATAACGATCTCTTTCAG | |
| *QC-Nde-rv* |  | CGTTATTGTTCATACGATATCTCCTGTGTGAAATTG | |
|  |  |  |  |
| pPT_roGFP2 expression plasmid | |  |  |
| *roGFP2_NdeI-fwd* |  | AACCCCATATGGTGAGCAAGGGCGAGGA | |
| *roGFP-_EcoR -rev* |  | GGGGGGAATTCTTACTTGTACAGCTCGTC | |
|  |  |  |  |
| pLK9 (pPT_roGFPiL expression plasmid) | |  |  |
| *roGFP-iL_XhoI-fwd* | | AAACTCGAGAAAAAGGAGAAGAACTTTTC | |
| *roGFP-iL_EcoRI-rev* | | AAAGAATTCTTATTTGTATAGTTCATCCATGC | |
|  |  |  |  |
| pLK16 (p416TFE_roGFP2_*Ec*DsbAΔSP expression plasmid) | |  |  |
| *DsbA*Δ*SP_EcoRI-fwd* | | AAAGAATTCGCGCAGTATGAAGATG | |
| *DsbA*Δ*SP_HindIII-fwd* | | TTTAAGCTTTTATTTTTTCTCGGACAGA | |
|  |  |  |  |
| *gshA* removal | |  |  |
| *gshA_fwd* | | AAATGTGTCTGTTAGCGGGATGGATGC | |
| *gshA_rev* | | AAAAGGCGCTTCCATCCGGGTATGATC | |
|  |  |  |  |
| Δ*dsbA* insertion | |  |  |
| *dsbA_fwd* |  | AAATTTACGCGCCATGCGTTTGGTTT | |
| *dsbA_rev* | | AAATTACGGCTAACGCAACAATAACAC | |
|  |  |  |  |
| KEIO primers (Baba et al., 2006) | |  |  |
| *k1* |  | CAGTCATAGCCGAATAGCCT | |
| *k2* |  | GGTGCCCTGAATGAACTGC | |
|  |  |  | |


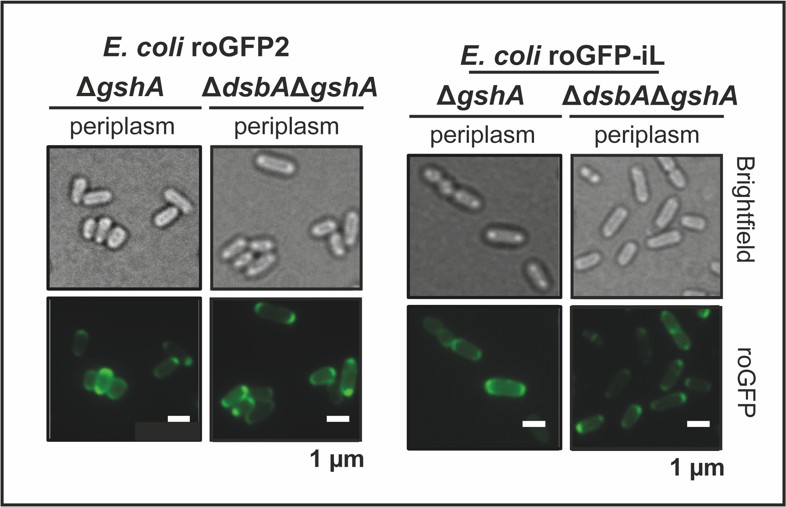


**Supplementary figure 1.** **roGFP probes localize in the periplasm of cells lacking GshA or DsbA and GshA.** Fluorescence microscopy of *E. coli* Δ*gshA* and Δ*dsbA*Δ*gshA* expressing roGFP2 (left) or roGFP-iL (right) confirming periplasmic localization of both roGFP probes.
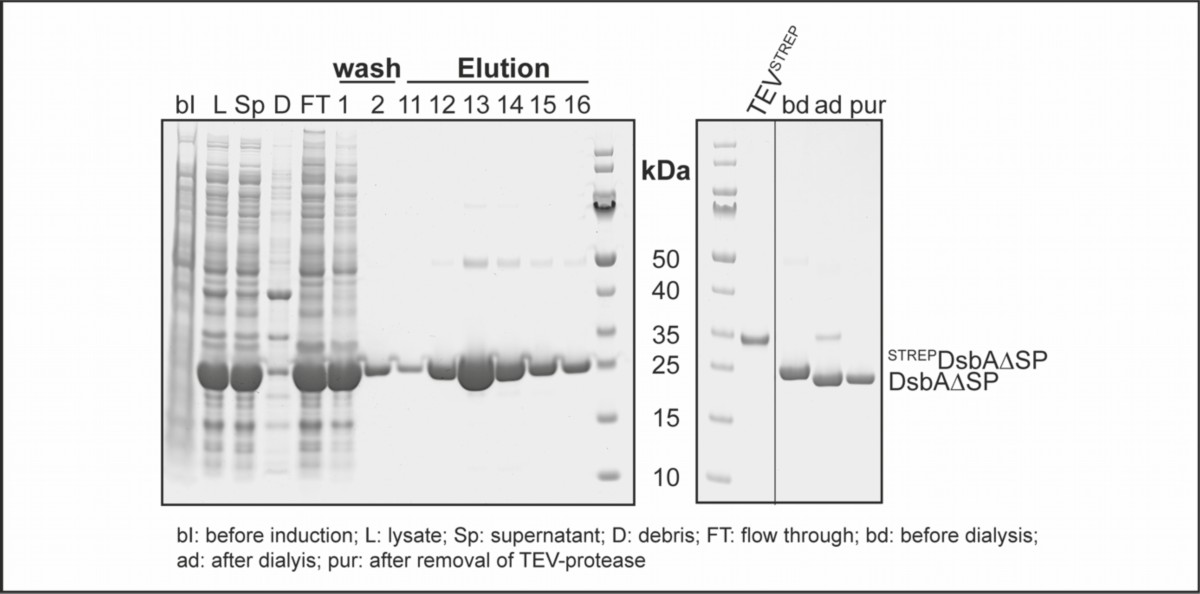


**Supplementary figure 2. Purification of tag-free DsbAΔSP from *E. coli* BL21 cell lysate**. SDS-PAGE of samples collected during the purification process. Cells harboring the plasmid for expression of ^STREP^DsbAΔSP were grown to an OD_600_ of 0.6-0.8 (bI) before expression was induced for 20 h at 20 °C. Then cells were harvested and lysed (L) and the lysate was separated by centrifugation (30 min, 4 °C, 25.000 x g) into supernatant (Sp) and debris (D). The supernatant was loaded onto a Streptavidin column (StrepTrap^TM^, GE Healthcare, Chicago, USA), washed with 5 CV buffer W (100 mM Tris-HCl, 150 mM NaCl, 1 mM EDTA, pH 8.0) and eluted with buffer E (100 mM Tris-HCl, 150 mM NaCl, 1 mM EDTA, 2.5 mM Desthiobiotin, pH 8.0) with the help of ÄKTApurifier (GE-Healthcare, Chicago, USA). Elution fractions were incubated over night at 4 °C during dialysis to buffer W with Strep-tagged TEV-protease (1:20 TEV^STREP^: ^STREP^DsbAΔSP) to get tag-free DsbA. The TEV-protease and uncleaved ^STREP^DsbAΔSP were removed using a Streptavidin column resulting in pure DsbAΔSP (pur).


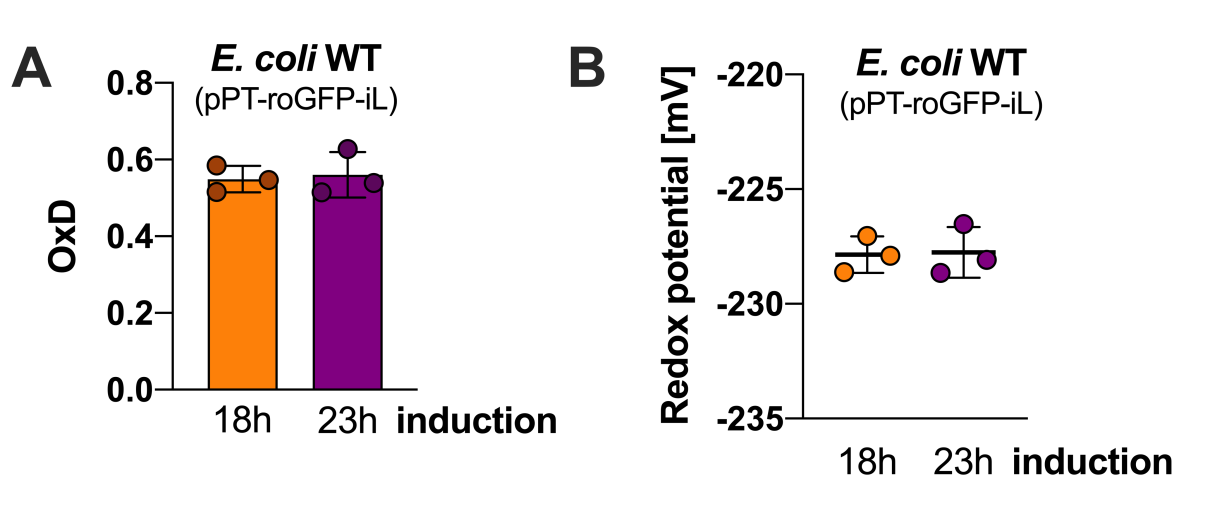


**Supplementary figure 3. Oxidation state and steady state redox potential of roGFP-iL in the periplasm of *E. coli* WT after 18 or 23 h of induction.** **(A)** Oxidation state (OxD) of roGFP-iL in the periplasm of *E. coli* WT. **(B)** The roGFP-iL steady state redox potential was calculated using the Nernst equation. Values were recorded in independent measurements and error bars represent the standard deviation in n=3 individual replicates.


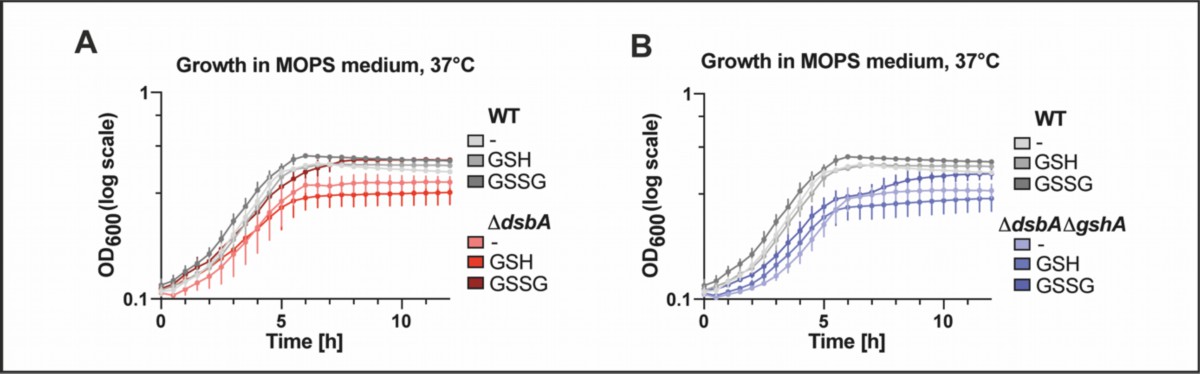


**Supplementary figure 4. Supplementation of the growth medium with oxidized glutathione rescues growth of DsbA-deficient cells, while GSH has an inhibitory effect.** *E. coli* WT **(A and B)**, Δ*dsbA* **(A)** and Δ*gshA*Δ*dsbA* **(B)** were cultivated at 37 °C in MOPS minimal medium without or with supplementation of 5 mM GSH or GSSG. OD_600_ was recorded over time and for better visualization y-axis is shown in log scale. Presented values are the mean of three biological replicates recorded in duplicate assays and error bars represent the standard deviation.
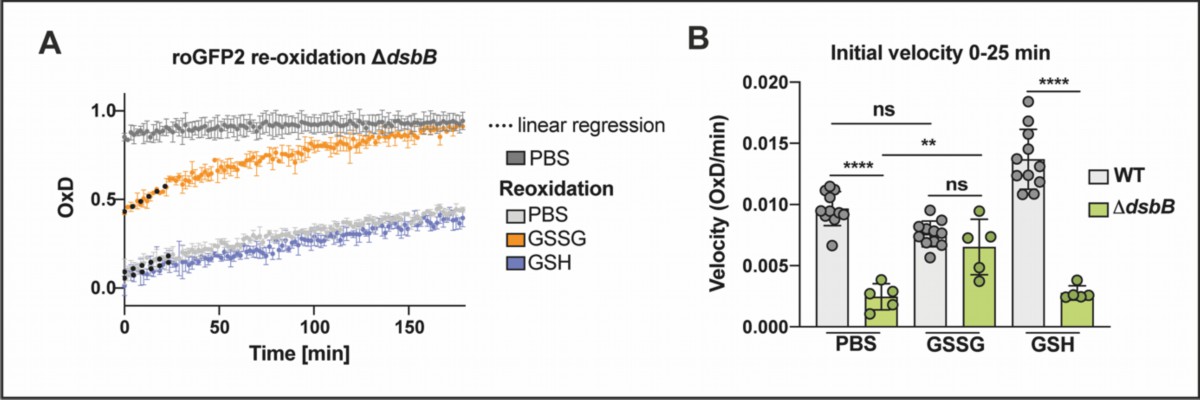


**Supplementary figure 5. External addition of oxidized glutathione rescues the reoxidation of roGFP2 in the periplasm of cells lacking DsbB.** **(A)** Reoxidation of periplasmic roGFP2 in *E. coli* Δ*dsbB*. Periplasmic reoxidation assay of roGFP2 in *E. coli* Δ*dsbB* was carried out as described in Fig. 3. One representative example out of at least five individual repeats is shown. Error bars represent standard deviation of technical triplicates. **(B)** The initial reoxidation velocity was calculated from linear regression in the first 25 min (dashed lines). Values (circles) are the mean of three technical replicates recorded in a minimum of five independent repeats. Error bars represent the standard deviation. Significance test was performed using one way ANOVA. ***p*<0.01, *****p* < 0.0001.

Baba, T., Ara, T., Hasegawa, M., Takai, Y., Okumura, Y., Baba, M., Datsenko, K.A., Tomita, M., Wanner, B.L., Mori, H., 2006. Construction of *Escherichia coli* K-12 in-frame, single-gene knockout mutants: the Keio collection. Molecular Systems Biology 2, 2006.0008. https://doi.org/10.1038/msb4100050

Blattner, F.R., Plunkett, G., Bloch, C.A., Perna, N.T., Burland, V., Riley, M., Collado-Vides, J., Glasner, J.D., Rode, C.K., Mayhew, G.F., Gregor, J., Davis, N.W., Kirkpatrick, H.A., Goeden, M.A., Rose, D.J., Mau, B., Shao, Y., 1997. The Complete Genome Sequence of *Escherichia coli* K-12. Science 277, 1453–1462. https://doi.org/10.1126/science.277.5331.1453

Degrossoli, A., Müller, A., Xie, K., Schneider, J.F., Bader, V., Winklhofer, K.F., Meyer, A.J., Leichert, L.I., 2018. Neutrophil-generated HOCl leads to non-specific thiol oxidation in phagocytized bacteria. eLife 7, e32288. https://doi.org/10.7554/eLife.32288

Lohman, J.R., Remington, S.J., 2008. Development of a family of redox-sensitive green fluorescent protein indicators for use in relatively oxidizing subcellular environments. Biochemistry 47, 8678–8688. https://doi.org/10.1021/bi800498g

Masuch, T., Kusnezowa, A., Nilewski, S., Bautista, J.T., Kourist, R., Leichert, L.I., 2015. A combined bioinformatics and functional metagenomics approach to discovering lipolytic biocatalysts. Frontiers in Microbiology 6. https://doi.org/10.3389/fmicb.2015.01110

Müller, A., Schneider, J.F., Degrossoli, A., Lupilova, N., Dick, T.P., Leichert, L.I., 2017. Systematic *in vitro* assessment of responses of roGFP2-based probes to physiologically relevant oxidant species. Free Radical Biology and Medicine 106, 329–338. https://doi.org/10.1016/j.freeradbiomed.2017.02.044

Studier, F.W., Moffatt, B.A., 1986. Use of bacteriophage T7 RNA polymerase to direct selective high-level expression of cloned genes. Journal of Molecular Biology 189, 113–130. https://doi.org/10.1016/0022-2836(86)90385-2

Taylor, R.G., Walker, D.C., Mclnnes, R.R., 1993. *E. coli* host strains significantly affect the quality of small scale plasmid DNA preparations used for sequencing. Nucleic Acids Research 21, 1677–1678. https://doi.org/10.1093/nar/21.7.1677

Zimmermann, J., Oestreicher, J., Geissel, F., Deponte, M., Morgan, B., 2021. An intracellular assay for activity screening and characterization of glutathione-dependent oxidoreductases. Free Radical Biology and Medicine 172, 340–349. https://doi.org/10.1016/j.freeradbiomed.2021.06.016
